# Challenges in diagnosing and managing sarcomatoid urothelial carcinoma of the renal pelvis: a case report

**DOI:** 10.3389/fonc.2025.1480790

**Published:** 2025-02-27

**Authors:** Jian Lei, Wei Zhao, Tao He, Hui Huang, Huayong Jian, Mei Zhang, Xike Luo, Xiaochuan Gong, Yan Wang

**Affiliations:** ^1^ Department of Urology, The Third Affiliated Hospital of Zunyi Medical University (The First People’s Hospital of Zunyi), Zunyi, China; ^2^ Department of Pathology, The Third Affiliated Hospital of Zunyi Medical University (The First People’s Hospital of Zunyi), Zunyi, China; ^3^ Department of Obstetrics, The Third Affiliated Hospital of Zunyi Medical University (The First People’s Hospital of Zunyi), Zunyi, China

**Keywords:** renal pelvis sarcomatoid urothelial carcinoma, rare disease, posterior laparoscopic radical nephrectomy for renal cancer, pathology, diagnosis, prognosis

## Abstract

Sarcomatoid urothelial carcinoma of the renal pelvis is an extremely rare malignant tumor with a high risk of recurrence and metastasis and a poor prognosis. This case reports a 61-year-old male patient with renal pelvic sarcomatoid urothelial carcinoma who developed extensive lymph node metastasis 26 days after posterior laparoscopic radical nephrectomy for renal carcinoma, and the patient died on the 45th postoperative day, with the cause of death being advanced malignancy of the tumor. This case highlights the rapid progression of sarcomatoid urothelial carcinoma, and sarcomatoid variants should be identified as early as possible, with active multidisciplinary adjuvant therapy and closer follow-up when feasible. Retrospective analysis of this patient’s treatment regimen and admission provides lessons for recognizing and aggressively managing this rare and fatal variant of urothelial carcinoma.

## Introduction

Urothelial carcinoma (UC) is the most common malignant tumor of the urinary system, most frequently occurring in the bladder, referred to as urinary bladder urothelial carcinoma (UBUC). Urothelial carcinoma of the ureter and renal collecting system is collectively known as upper tract urothelial carcinoma (UTUC), which is relatively rare, accounting for approximately 5-10% of all urothelial carcinomas. Renal pelvis sarcomatoid carcinoma (RPSC) is an extremely rare variant of urothelial carcinoma, composed of both epithelial and stromal components, characterized by high malignancy and poor prognosis (1). It is estimated that the annual incidence is 1-2 cases per 100,000 people (2). Due to its rarity, there is currently no consensus or recommendations regarding the treatment of renal pelvis sarcomatoid urothelial carcinoma. At this stage, radical nephroureterectomy is considered the standard treatment for non-metastatic high-risk UTUC. Here, we report a case of a 61-year-old male patient with renal pelvis sarcomatoid urothelial carcinoma who developed extensive lymph node metastasis 26 days after undergoing laparoscopic radical nephrectomy. The patient did not receive chemotherapy, immunotherapy, or targeted therapy as adjuvant treatment and succumbed to the disease on postoperative day 45, with the cause of death attributed to advanced cancer cachexia. Here, we report a case of a 61-year-old male patient who was initially suspected of having renal cancer and underwent retroperitoneal laparoscopic radical nephrectomy. The postoperative pathological diagnosis was renal pelvic sarcomatoid urothelial carcinoma. The patient developed extensive lymph node metastasis 26 days post-surgery, without having received adjuvant therapies such as chemotherapy, immunotherapy, or targeted therapy. The patient succumbed to advanced cancer cachexia on day 45 post-surgery.

## Case report

The patient is a 61-year-old male who was admitted to our hospital on March 27, 2024, due to hematuria for one month and right flank pain lasting over ten days. Previously in good health condition. He has a history of smoking for over 30 years, averaging 20 cigarettes per day, and has quit smoking for two years. He has also been consuming alcohol for over 30 years, averaging 100-150 mL per day, and has abstained from alcohol for the past three months. There is no family history of tumors.

On physical examination, there was percussion pain in the right renal region and no noticeable masses were palpated in the right kidney region. Other examinations did not reveal significant abnormalities. Urine analysis showed abnormal white blood cells and red blood cells, while other laboratory tests, including complete blood routine and blood biochemistry, showed no significant abnormalities. The CT scan from an outside hospital suggested a right kidney mass with uneven soft tissue density, right renal pelvis stones, and fluid accumulation, as well as multiple stones in the right kidney.

**Figure 1:**
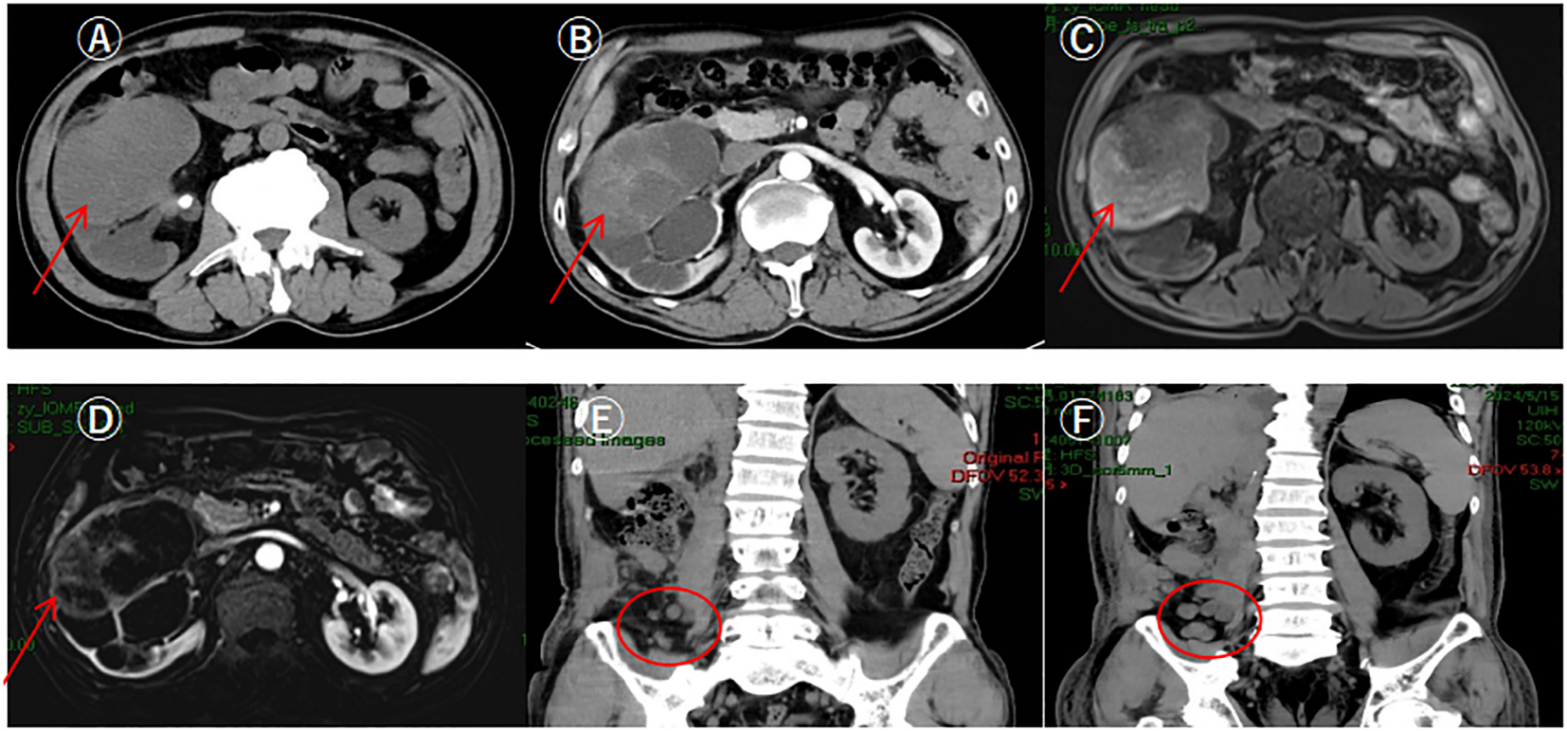
Patient's preoperative CT urography, upper abdominal MRI and postoperative upper abdominal CT. **(A)** CTU showed a huge soft tissue mass shadow in the middle part of the right kidney, with clear margins of the mass, and its inner density was uneven (pointed out by arrows); **(B)** CTU enhancement scan showed a mild uneven delayed enhancement, with a slight thickening of the neighboring fascia, and gross; **(C)** MRI showed a mass shadow in the mid-pole of the right kidney, with an uneven inner signal, and clear boundaries; **(D)** MRI enhancement scan showed uneven enhancement; **(E)** Review CT on the 26th day after surgery showed multiple round-like soft tissue nodules in the right iliac fossa, with a diameter of about 1.0 cm, and lymph node metastasis was considered; **(F)** Review CT on the 37th day after surgery showed multiple nodular and irregular mass-like soft tissue density shadows in the operated area and the right posterior abdominal wall and the right iliac fossa, with the foci enlarging and increasing in size compared with the previous ones, and lymph nodes were considered to be multiple metastasis.

After admission, a CT urogram was performed, indicating a right renal mass: a neoplastic lesion needs to be ruled out, along with multiple stones in the right kidney, and stones at the right renal pelvis-ureter junction causing hydronephrosis (**Figure 1**). SPECT renal dynamic imaging suggested: split renal GFR: 56.2 ml/min in left kidney, 28.8 ml/min in right kidney. An MRI of the upper abdomen showed stones at the right renal pelvis-ureter junction with hydronephrosis and a mass in the mid-polar region of right kidney, considering renal cancer (**Figure 1**).

**Figure 2:**
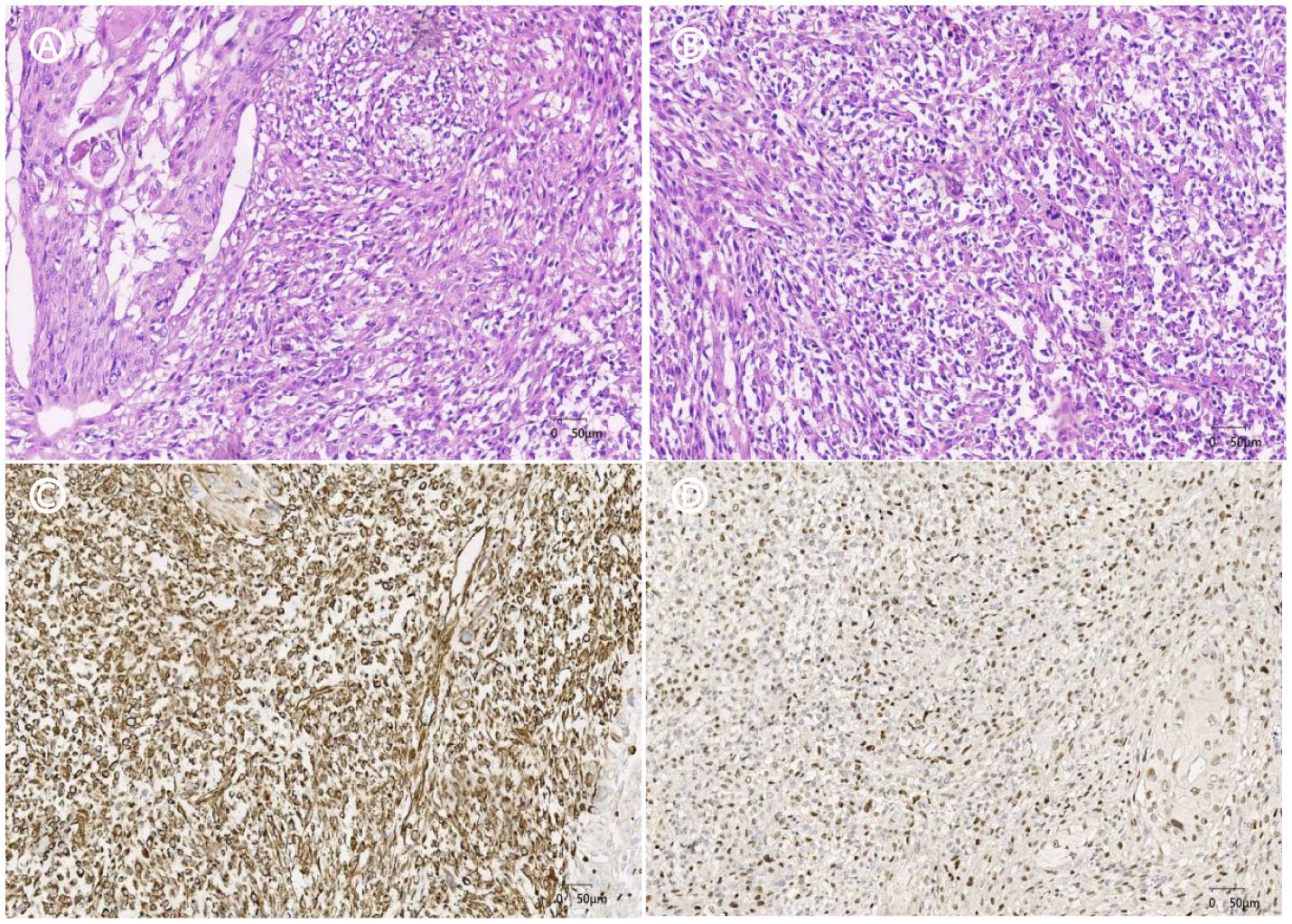
HE staining and immunohistochemistry **(A)**. HE×20, the tumor consisted of a mixture of high-grade uroepithelial carcinoma (left) and sarcomatoid component; **(B)**. HE×20, the sarcomatoid component cells were spindle-shaped and polygonal, with obvious cellular anisotropy, mitotic count was easy to be seen, and multinucleated giant cells were seen; **(C)**. IHC×20. Vimentin sarcoma-like region positive for tumor cells; **(D)**.IHC×20, the tumor cells in the sarcomatoid region of GATA-3 were positive.

After excluding surgical contraindications, a retroperitoneal laparoscopic radical nephrectomy was performed under general anesthesia. During the procedure, the right kidney and the proximal half of the ureter were removed. Postoperative pathological examination revealed that the right kidney had significantly enlarged, measuring approximately 15cm × 9cm × 6cm. In the renal pelvis section, a mass approximately 6cm × 4.5cm × 4cm in size was observed. The mass was grayish-white, solid, moderately textured, and had indistinct boundaries with the surrounding tissues. Microscopic examination revealed that the tumor was composed of spindle-shaped cells arranged in a fascicular or haphazard pattern, with an interstitial and myxoid stroma and scattered multinucleated giant cells, mitotic count were observed (**Figure 2**). Immunohistochemical staining suggested Vimentin (+), GATA-3 (+), CK20 (+), CK8/18 (+), CD10 (partially +), EMA (+), P40 (+), P60 (+), Pax-8 (-), and Ki-67 (+, about 60%) (**Figure 2**).

Postoperative pathological diagnosis: sarcomatoid uroepithelial carcinoma of the right renal pelvis with massive hemorrhage and necrosis, the tumor size was about 6.0cm×4.5cm×4.0cm, there was no obvious vasculature and nerve invasion, and there was no tumor involvement in ureteral dissection, renal peritoneum, renal hilar blood vessel dissection, and perirenal adipose tissue. Although the surgery was successful, the patient’s postoperative follow-up revealed lymph node metastasis (**Figure 1**). Chemotherapy and other adjuvant treatments were recommended to the patient and their family, but they declined. The patient passed away on the 45th postoperative day due to late-stage cachexia related to the tumor.

## Discussion

A middle-aged male patient presented with initial symptoms of hematuria and flank pain, and was found to have a space-occupying lesion in the right kidney, with no family history of malignancy. Based on histological, immunohistochemical, and imaging examination results, the patient was diagnosed with right renal pelvic carcinoma with sarcomatoid differentiation, with a tumor stage of T_1_N_0_M_0_.Uroepithelial carcinoma is mainly originated from the epithelial cells of the urothelium, and most of them are found in the bladder, but about 25% of the patients contain various subtypes such as variant histology (VH), which includes various subtypes such as squamous differentiation, adenoid differentiation, neuroendocrine differentiation, and sarcomatoid differentiation. Pure variant histology types are very rare and account for only 5% of upper urinary tract tumors (3).Malignant tumors occurring in the renal pelvis are most commonly associated with migratory cell carcinoma and according to Mousavi SE et al, the incidence of renal pelvis carcinoma increases with age, especially after the age of 55 years where the increase in incidence is more pronounced, and peaks in the age group of 80 - 84 years for both males and females, with both males having a higher incidence than females (4). The more common symptoms of clinical presentation are painless hematuria, pain and palpable mass and the common complications associated with it include kidney stones (5, 6). In our case, the patient presented with first symptoms of hematuria in the flesh and pain in the lower back, initial examination revealed multiple stones in the patient’s right kidney, and further examination revealed a tumor in the right kidney, which is in general agreement with the thesis of previous studies. The EAU guidelines suggest that smoking and aristolochic acid increase the risk of UTUC, with smoking being the main risk factor for UC. Studies of UTUC have estimated an increase in relative risk from 2.5% to 7%, Aristolochic acid is the active ingredient in the herbaceous plant Aristolochiaceae. Its accidental ingestion and traditional medication also increase the risk of UTUC (5, 7). As mentioned earlier, pyelosarcoma-like, a rare variant histologic subtype of uroepithelial carcinoma, is a high-risk uroepithelial carcinoma that has a significantly lower median survival compared to conventional UTUC. It also has a high risk of recurrence and metastasis and a very poor prognosis (3, 5, 8). In our case, extensive lymph node metastasis (right posterior abdominal wall, diaphragmatic pedicle, paraspinal erector spinae muscle, and right iliac fossa lymph node metastasis) appeared on 26 days postoperatively, and the patient died on 45 days postoperatively due to advanced malignancy of the tumor.

The imaging manifestation of renal pelvis sarcomatoid carcinoma lacks specificity, CT urography is the most effective and widely used imaging modality with the highest diagnostic accuracy, which usually manifests as an irregular mass in the renal pelvis with blurred borders and foci of necrosis and hemorrhage, and the enhancement scans show obvious enhancement of the surrounding normal renal parenchyma and mild enhancement of the lesion, and the delayed scans show the change of filling defects in the pelvis in the area of the lesion. In delayed scanning, filling defects can be seen in the renal pelvis of the focal area. It may also invade the surrounding tissues of the kidney and neighboring organs. Magnetic resonance imaging (MRI) can show a substantial renal pelvic space, and the signal is often heterogeneous, with a low signal on T1-weighted imaging and a high signal on T2-weighted imaging. Positron emission tomography/computed tomography (PET/CT) has high diagnostic value in evaluating lymph node and distant metastasis of renal pelvic uroepithelial cancer (9). In addition, the development of liquid biopsy technology also provides valuable information for the diagnosis of UTUC. Liquid biopsy can analyze various tumor-derived components in urine, such as circulating tumor cells, cfDNA, cfRNA, proteins, and metabolites, and the combined application of multiple markers can improve the accuracy of diagnosis (10). The definitive diagnosis of renal pelvic sarcomatoid carcinoma relies on histopathology, and the tumor cells are biphasic, with both uroepithelial and sarcomatoid components. The nuclear heterogeneity of the tumor cells is obvious, and a large number of mitotic figures are common, and multinucleated giant cells, necrosis, and lymphovascular invasion may also be seen (11, 12). It needs to be differentiated from carcinosarcoma, sarcomatoid renal carcinoma, and pelvic uroepithelial carcinoma, which contain both epithelial and sarcomatoid components, while sarcomatoid carcinoma mainly originates from the epithelial component, where the sarcomatoid component is a metaplasia of cancerous tissue. Since carcinosarcoma and sarcomatoid carcinoma have a similar appearance under the microscope (13),immunohistochemistry is important in the differentiation of these two tumors, and the epithelial component is usually positive for CK and EMA. The mesenchymal component, on the other hand, tends to present vimentin positivity (14). Immunohistochemistry in our patient confirmed CK20 (+), CK8/18 (+), EMA (+), GATA-3 (+), and vimentin (+), and the patient’s Ki-67 (+, about 60%), and CD10 (partially +) indicated that the tumor was highly malignant and aggressive.

There is no uniform view on the treatment of pelvic sarcomatoid uroepithelial carcinoma, and radical nephroureterectomy is the standard treatment for non-metastatic high-risk UTUC in general with reference to renal pelvic tumors (15, 16). However, since the disease is commonly seen in the elderly population, whose renal function is already impaired by aging and other comorbidities, radical resection has a negative impact on overall renal function. According to Saini S et al, in patients with low-risk, limited UTUC, surgery with preservation of the renal unit can be considered (17), but the recurrence rate is between 36-54%, and the EAU has stated that endoluminal application of mitomycin C is a well-tolerated, feasible and potentially beneficial treatment for low-risk UTUC, but the efficacy of existing modalities such as percutaneous nephrostomy tubes for downstream administration and ureteral stents for inducing urinary reflux of the bladder to administer the drug is less than optimal, and how to deliver the drug efficiently up the urinary tract is the difficulty of endoluminal therapy (18). In one study, UTUC patients treated with a combination of neoadjuvant gemcitabine and cisplatin had a postoperative residual rate of 37.5 per cent, compared with only 12.5 per cent in the surgery-only group. Another study showed that patients receiving a combination of neoadjuvant avelumab and neoadjuvant chemotherapy had an objective remission rate of 45.8% compared to 12.5% in the surgery-only group. The results of the CheckMate 274 study showed that in a cohort of 21% (149/709) of UTUC patients, immune checkpoint inhibitor monotherapy as a high-risk postoperative treatment for patients at high risk after radical surgery adjuvant therapy in patients at risk of recurrence significantly improved disease-free survival (19). Overall, NAC has some potential benefit in UTUC, and patients without NAC had shorter disease-free survival relative to those who received NAC. Regarding the management of hilar renal tumors, research by Savio Domenico Pandolfo et al. demonstrates that robotic-assisted partial nephrectomy (RAPN) offers several advantages over conventional open surgery. These include smaller incisions, improved visualization, enhanced precision, faster postoperative recovery, and comparable complication rates. The implementation of novel suturing and clamping techniques, along with individualized management strategies for hilar tumors, facilitates optimal tumor control and minimizes impact on renal function, ultimately improving patient survival. While RAPN represents an effective approach for treating hilar renal tumors, personalized treatment planning remains crucial for ensuring surgical success (20). For metastatic UTUC, the NCCN Clinical Practice Guidelines in Oncology recommend platinum-based drugs and immune checkpoint inhibitors for chemotherapy with the aim of achieving better treatment outcomes. Studies have shown that compared to chemotherapy alone, chemotherapy combined with nephroureterectomy resulted in a median survival time of 25 months, which was significantly longer than that of patients who received chemotherapy alone (7, 8 months) and reduced the risk of death (21). Neoadjuvant chemotherapy may be associated with improved pathologic downstaging and complete response rates post-surgery, while decreasing the risk of recurrence and mortality, and enhancing the resectability and prognosis of patients with locally advanced upper tract urothelial carcinoma. Although adjuvant chemotherapy following surgery has been demonstrated to reduce the risk of recurrence after radical nephroureterectomy (22),for sarcomatoid urothelial carcinoma of the renal pelvis, while chemotherapy can offer temporary symptomatic relief, immunotherapy is regarded as a more promising treatment option to enhance patient prognosis (23). Regarding whether lymph node dissection is performed in the treatment of UTUC, for high-risk non-metastatic upper urinary tract urothelial carcinoma patients, performing regional lymph node dissection can achieve a better long-term prognosis (5), but others have suggested that the need for lymph node dissection remains controversial in patients without clinically significant lymph node metastases (24). Studies have shown that lymph node metastasis is an independent predictor of mortality in UTUC patients (25), lymph node metastasis means that the cancer cells have spread to other parts of the body, and the condition is more serious, and more aggressive treatment measures should be taken to improve their overall survival rate. Due to the high expression of PD-L1 in sarcomatoid carcinoma, a patient with renal pelvic sarcomatoid uroepithelial carcinoma underwent immunotherapy with a combination of nivolumab and ipilimumab. As a result, most of the lesions shrank. However, due to bleeding from the left kidney and recurrent bladder congestion, the patient had to undergo a left nephrectomy and para-aortic lymph node dissection. Two months post-surgery, multiple new lung metastases appeared. The patient was then treated with the chemotherapy drugs gemcitabine and cisplatin, followed by the immunotherapy drug pembrolizumab. Despite these treatments, the patient’s condition progressed, and they ultimately succumbed five months after the surgery (23). Another 81-year-old male patient with renal pelvis sarcomatoid urothelial carcinoma experienced tumor recurrence and invasion of the retroperitoneum and inferior vena cava 4.5 months after undergoing total nephrectomy. Following immunotherapy with Tislelizumab, a suspected “pseudoprogression” phenomenon was observed. When combined with Anlotinib targeted therapy, partial clinical remission was sustained for 20 months, accompanied by significant tumor shrinkage and symptom improvement (26). These cases indicate that actively receiving immunotherapy and targeted therapy has potential therapeutic effects on metastatic renal pelvis sarcomatoid urothelial carcinoma, which can delay disease progression and prolong patient life expectancy.

## Conclusion

In summary, we report a case of renal pelvis sarcomatoid urothelial carcinoma. The patient was diagnosed with renal cell carcinoma following preoperative examination, with no obvious signs of metastasis observed. He underwent retroperitoneal laparoscopic radical nephrectomy, which proceeded without complications. The postoperative pathological diagnosis confirmed renal pelvis sarcomatoid urothelial carcinoma. Unfortunately, the patient developed widespread metastases 26 days after the surgery and succumbed to the disease 45 days post-surgery due to progression. Given the high invasiveness and propensity for early metastasis of renal pelvis sarcomatoid urothelial carcinoma, coupled with its poor prognosis, early diagnosis and the development of personalized treatment plans through multidisciplinary team collaboration are essential for enhancing patients’ survival rates and quality of life.

## Data Availability

The raw data supporting the conclusions of this article will be made available by the authors, without undue reservation.
