# Supplementary figures and images for: Challenges in diagnosing and managing sarcomatoid urothelial carcinoma of the renal pelvis: a case report
Source: Front Oncol. 2025 Feb 27;15:1480790. doi: 10.3389/fonc.2025.1480790 (PMC11903244; doi:10.3389/fonc.2025.1480790)

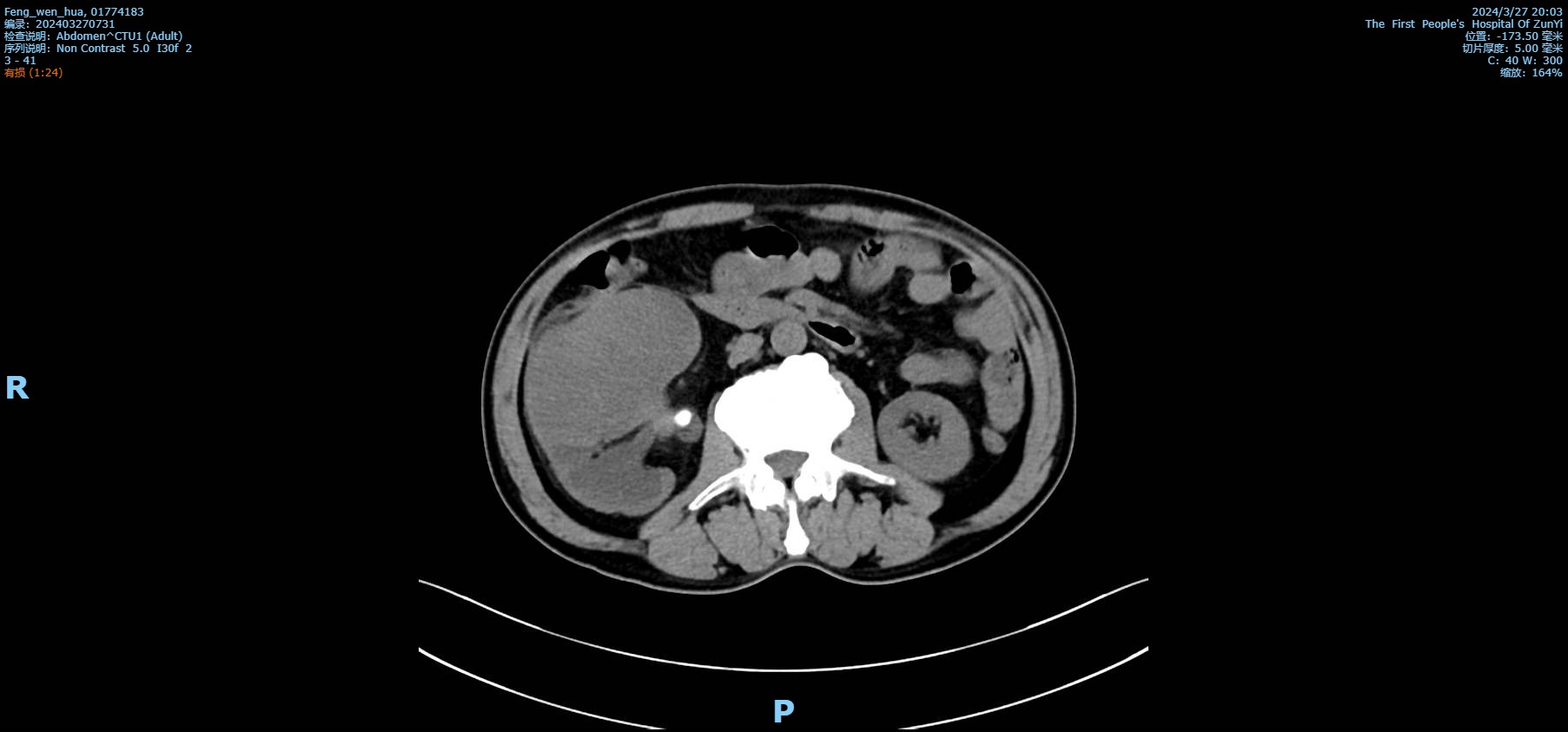

Supplement: Supplementary file 1 [file Image1.jpeg]

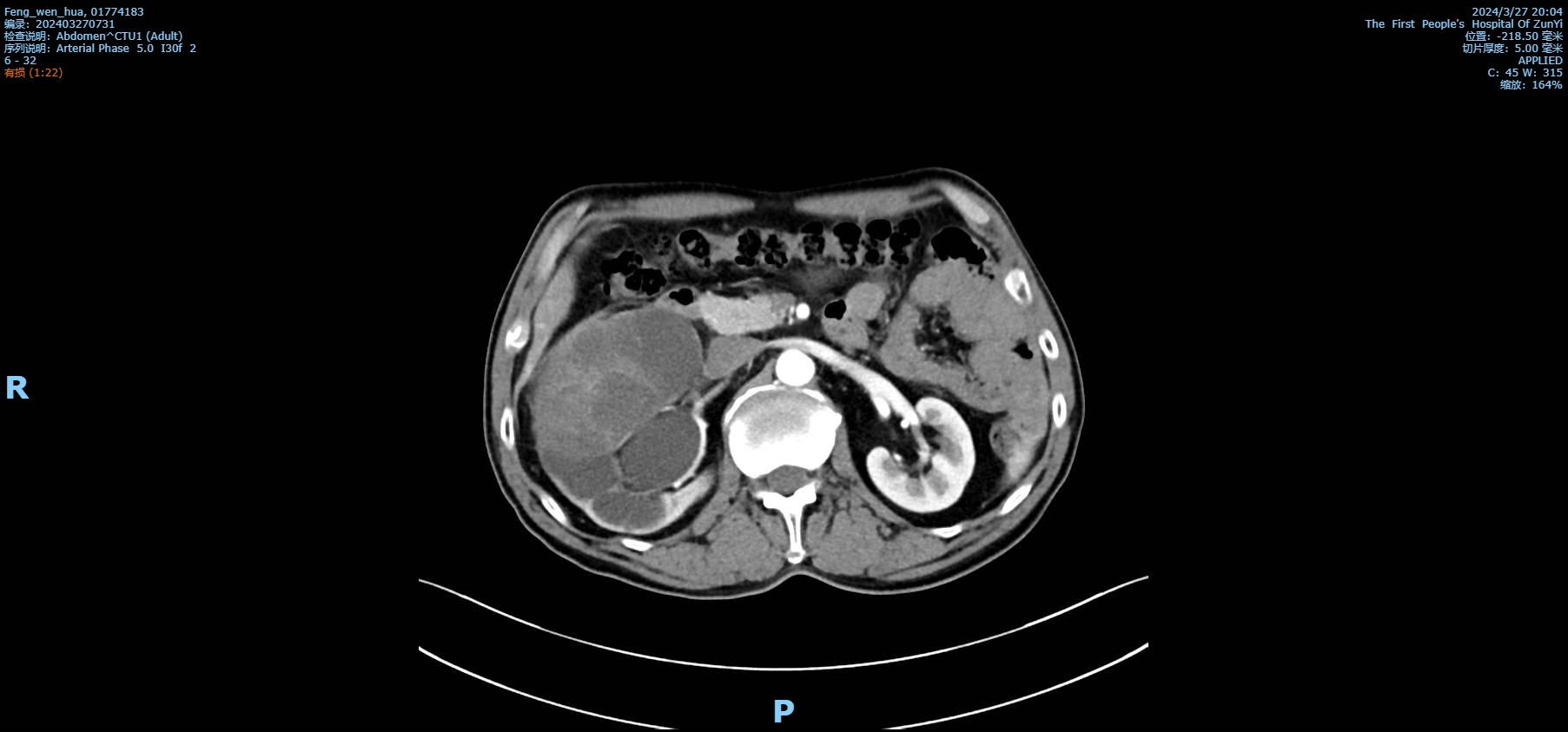

Supplement: Supplementary file 2 [file Image2.jpeg]

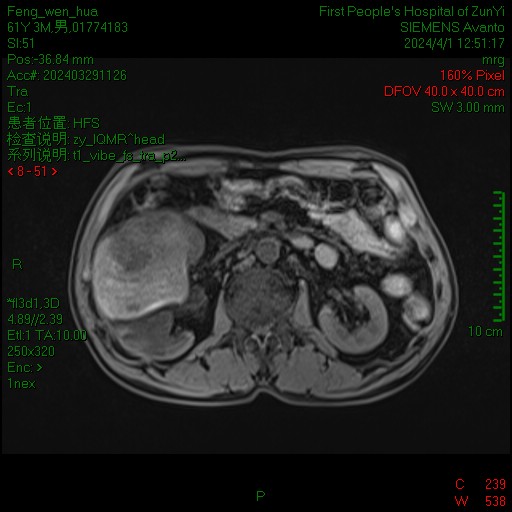

Supplement: Supplementary file 3 [file Image3.jpeg]

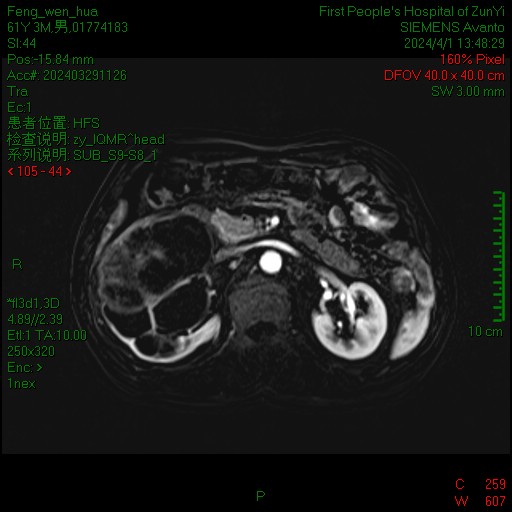

Supplement: Supplementary file 4 [file Image4.jpeg]

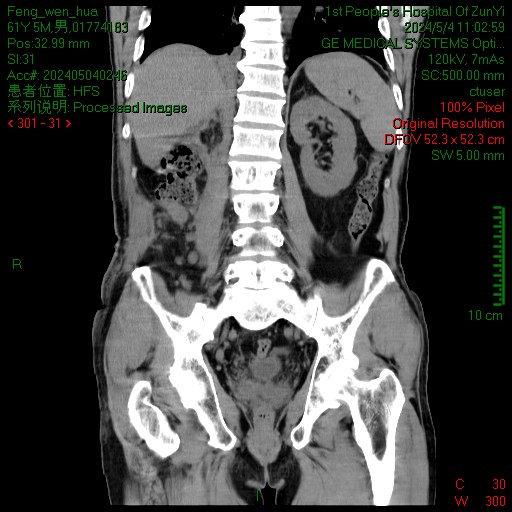

Supplement: Supplementary file 5 [file Image5.jpeg]

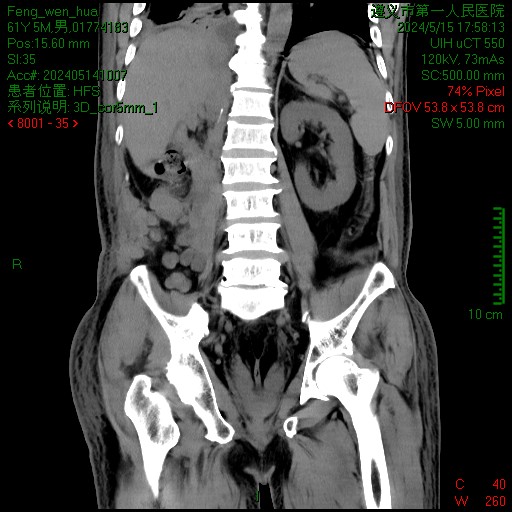

Supplement: Supplementary file 6 [file Image6.jpeg]
